# Supplementary material for: Social and nonsocial synchrony are interrelated and romantically attractive
Source: Commun Psychol. 2024 Jun 10;2:57. doi: 10.1038/s44271-024-00109-1 (PMC11332061; doi:10.1038/s44271-024-00109-1)
Supplement: Supplementary file 2 — Supplementary Information [file 44271_2024_109_MOESM2_ESM.pdf]

Social and nonsocial synchrony are interrelated and romantically attractive

Cohen M., Abargil M., Ahissar M., & Atzil S.

Supplementary Results

Supplementary Note S1: Behavioral analyses of the stimuli in the online experiment

While preparing the stimuli, we deliberately controlled for elements in the video that could potentially impact the participants' ratings, including the interaction setup, the actors' positions, the conversation content, physical appearance, and the video length.

To further address potential differences between the two videos, we conducted post hoc behavioral analyses to characterize the behavioral display of the actors in the two videos. Specifically, we coded the actors’ facial expressions, body movements, gaze, and vocalization, using a second-by-second behavioral coding procedure commonly used in our lab (see Abu Salih et al., 2023 for a detailed method[1]). The physiological and behavioral differences between the two videos are summarized in Table S1 (from the largest to the lowest effect).

Table S1: Quantifying behavior and physiological arousal of the actor and actress in the synchronous and non-synchronous videos.

Variable	Synchronous video	Non-synchronous video	Ratio Synch/Non-synch (Absolute Value)
Physiological (EDA) man-woman synchrony (Pearson’s r)	0.614	-0.097	6.33
Social gaze woman (Score)	0.796	0.323	2.464
Facial expression man-woman synchrony (Score)	0.359	0.173	2.075

Social gaze man (Score)	0.763	0.527	1.448
Body movement woman (Score)	1.108	0.871	1.272
Facial expression man (Score)	2.366	2.194	1.078
Vocalization man (Score)	1.022	0.957	1.068
Vocalization woman (Score)	1.366	1.29	1.059
Facial expression woman (Score)	2.355	2.419	0.974
Body movement man (Score)	1.183	1.473	0.803
Vocalization man-woman synchrony (Score)	-0.312	-0.435	0.717
Body movement man-woman synchrony (Score)	0.169	-0.241	0.701
Physiological arousal (EDA) woman (Score)	0.388	0.961	0.404
Physiological arousal (EDA) man (Score)	0.969	3.083	0.314
Social gaze man-woman synchrony (Score)	-0.031	-0.129	0.24

*The Ratio is calculated using the correlation coefficients' absolute values to account for the mutual change between the in-phase and anti-phase over time[2].

This post hoc descriptive analysis reveals possible bio-behavioral facets that impact the raters' attraction detection. The analysis shows that the most significant difference between the two videos is the physiological synchrony between the man and woman; it increased six times in the synchronous video compared to the non-synchronous video. This provides a strong manipulation check to the experimental

paradigm. Additionally, synchronization in facial expressions is two times higher in the synchronous video than the non-synchronous video. Social gaze was slightly more evident in the synchronous video (2.46 times more social gaze in the woman and 1.45 times more social gaze in the man in the synchronous interaction). Last, the physiological arousal levels are lower in the synchronous video (3.18 times lower in the man and 2.48 times lower in the woman).

The behavioral differences between the videos can serve two functions in this experiment. The increased synchrony in facial expression and increased gaze by the woman can serve as visual cues for the raters, used to assess the attractiveness of the actors. Second, it can serve the actors to synchronize their physiology when instructed. Gaze was previously associated with higher synchrony in different processes, such as neural activity[3–5] and behavior[6]. However, increased gaze was tested as a marker of attraction and was not predictive of romantic interest on its own[7]. Likewise, in this study, lower arousal was not associated with attraction on its own (measured in the dating experiment, Spearman $r = -0.12$; $p = 0.343$; 95% confidence interval = [-0.407, 0.166]; $N = 64$). Importantly, this is a descriptive analysis on two videos. Future research is needed to investigate how people regulate their behavior in order to synchronize during social interactions, and how different behavioral patterns potentially serve as markers for romantic attraction.

The physiological and behavioral raw data in the two videos is depicted in Figure S1.

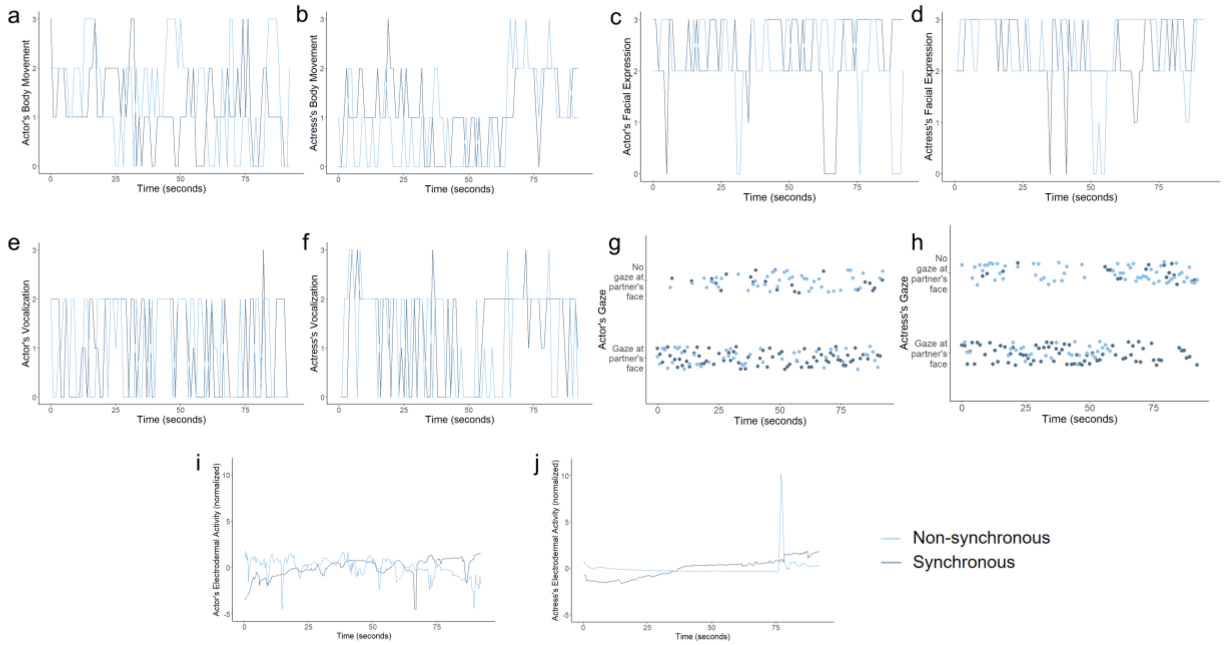

Figure S1. The physiological and behavioral measures of the actors in the synchronous and non-synchronous videos. The figure depicts the level of body movements, facial expressions, vocalization, gaze, and physiological arousal of the actor and actress in the synchronous and nonsynchronous videos. (a-b) The actors' body movement in the videos (0- no movement; 1- slight movement; 2- significant movement; 3- effortful body position with significant movement); (c-d) The actors' facial expression in the videos (0- no expression; 1- speaking with no expression; 2- mild expression; 3- significant expression); (e-f) The actors' vocalization in the videos (0- no vocalization; 1- low vocalization; 2- normal speech; 3- high or rapid speech or laugh); (g-h) The actors' gaze in the videos (0- not looking at the partner's face; 1- looking at the partner's face); (i-j) The actors' physiological arousal in the videos (Electrodermal activity). Behavioral values are coded separately to each partner for each second of the video, and represent the level of each behavior. The physiological arousal of the actors was normalized to account for differences in the Empatica electrodermal baseline and the position of the wristband. See Table S1 for descriptive statistics of all conditions. See Abu Salih et al. (2023) for a detailed explanation of the method[1].

Supplementary Note S2: Multiple exploratory measurements of electrodermal synchrony

Electrodermal synchrony can be computed with two approaches. The first is Pearson correlation between the partners' electrodermal activity since it reflects the in-phase mutual change over time. A second approach calculates the squared correlation to account for both the in-phase and anti-phase mutual change over time[2]. Moreover, synchrony can be calculated for the first two minutes of the date, as this was found to be most predictive of romantic interest[8], or for the entire date duration, which was hypothesized to represent the individual ability to synchronize mostly. The results show that social electrodermal synchrony is a stable measure that predicts both attraction and nonsocial synchrony, whether calculated for the entire date or the first two minutes. Moreover, in line with our hypotheses, *Individual Electrodermal Synchrony Scores* in the first two minutes showed the strongest correlation with *Individual Romantic Attractiveness Scores*[8]. In contrast, the correlation across the entire five minutes showed the strongest correlation with the *Individual Sensorimotor Synchrony* in the finger tapping task. In contrast to our hypothesis[8], the squared correlations did not strongly predict attraction. Here we included exploratory analyses of the multiple relevant options to calculate synchrony and accordingly applied Bonferroni correction for multiple hypotheses testing in each research question. The corrected p-value is 0.012, and the reported results remain significant.

S2.1 Initial motivation and electrodermal synchrony:

We conducted a multilevel model analysis on 64 dates, testing the extent to which initial attraction at the beginning of a date leads to increased synchrony during the date for each synchrony measure.

Synchrony during the first 2 minutes (not squared) and initial interest: men (β= 0.045, p= 0.474); women (β= 0.012, p= 0.791); N = 64 dates.

Synchrony during the entire date (not squared) and initial interest: men (β= 0.061, p= 0.44); women (β= 0.039, p= 0.513); N = 64 dates.

Analyses with the squared synchrony measurements did not converge in a multilevel model. The Spearman correlation values are reported here:

Synchrony during the first 2 minutes (squared) and initial mutual interest: *Spearman r* = 0.12; *p* = 0.343; *95% confidence interval* = [-0.126, 0.367]; N = 64 dates.

Synchrony during the entire date (squared) and initial mutual interest: *Spearman r* = 0.216; *p* = 0.086; *95% confidence interval* = [-0.031, 0.464]; N = 64 dates.

S2.2 Electrodermal synchrony and sensorimotor synchrony:

We computed the correlation between the individual social synchrony measurements and the individual sensorimotor synchrony scores (individual scores are averaged across multiple dates per participant).

Synchrony during the first 2 minutes (not squared) and sensorimotor synchrony: *Pearson r* = 0.432; *p* = 0.022; *95% confidence interval* = [0.07, 0.693]; N = 28.

Synchrony during the entire date (not squared) and sensorimotor synchrony: *Pearson r* = 0.494; *p* = 0.008; *95% confidence interval* = [0.148, 0.732]; N = 28.

Synchrony during the first 2 minutes (squared) and sensorimotor synchrony: *Pearson r* = 0.105; *p* = 0.597; *95% confidence interval* = [-0.279, 0.46]; N = 28.

Synchrony during the entire date (squared) and sensorimotor synchrony: *Pearson r* = 0.323; *p* = 0.094; *95% confidence interval* = [-0.057, 0.621]; N = 28.

S2.3 Electrodermal synchrony and attractiveness:

We computed the correlation between the individual social synchrony measurements and the individual romantic attractiveness scores (individual scores are averaged across multiple dates per participant).

Synchrony during the first 2 minutes (not squared) and romantic attractiveness: *Spearman r* = 0.415; *p* = 0.018; *95% confidence interval* = [0.08, 0.746]; N = 32.

Synchrony during the entire date (not squared) and romantic attractiveness: *Spearman r* = 0.342; *p* = 0.056; *95% confidence interval* = [-0.015, 0.695]; N = 32.

Synchrony during the first 2 minutes (squared) and romantic attractiveness: *Spearman r* = 0.172; *p* = 0.347; *95% confidence interval* = [-0.214, 0.556]; N = 32.

Synchrony during the entire date (squared) and romantic attractiveness: *Spearman r* = 0.214; *p* = 0.24; *95% confidence interval* = [-0.192, 0.618]; N = 32.

Supplementary Note S3: Analyses of the different synchrony groups

The distribution of individual electrodermal synchrony scores across all participants ranged from -0.316 to 0.692. Super Synchronizers were defined as the upper third of this distribution [range: 0.33, 0.692], Medium Synchronizers were defined as the middle third [range: 0.16, 0.311], and Low Synchronizers as the lower third [range: -0.316, 0.078]. Accordingly, the difference between the groups captures the variation in social synchrony scores: F(2, 29) = 70.36, p-value < 0.001, N = 32 (Low Synchronizers vs. Medium Synchronizers: p-value < 0.001, Cohen's d = 3.233, df = 15.873, t = -7.6; Low Synchronizers vs. Super Synchronizers: p-value < 0.001, Cohen's d = 4.427, df = 19.367, t = -10.381; Medium Synchronizers vs. Super Synchronizers: p-value < 0.001, Cohen's d = 2.358, df = 14.351, t = -5.574) (Figure S2a). The results show that some individuals better synchronize, regardless of the partner. These super synchronizers also better synchronize in the nonsocial task, and are considered more attractive.

When assessing the associations between social synchrony, nonsocial synchrony, and attractiveness separately for the different synchrony groups (Figure S3), we find that low social synchronizers (low social physiological synchrony) are significantly less synchronized in the nonsocial sensorimotor task than medium and super synchronizers: F(2, 25) = 6.658, p-value = 0.005, N = 28 (Low Synchronizers vs. Medium Synchronizers: p-value = 0.022, Cohen's d = 1.261, df = 11.453, t = -2.654; Low Synchronizers vs. Super Synchronizers: p-value = 0.016, Cohen's d = 1.353, df = 10.766, t = -2.87) (Figure S2b). When assessing the association between synchrony and attraction, we find that super social synchronizers (high social physiological synchrony) are rated as more attractive than low synchronizers: F(2, 29) = 4.64, p-value = 0.018, N = 32 (Low Synchronizers vs.

Super Synchronizers: p-value = 0.008, Cohen's d = 1.246, df = 19.916, t = -2.923) (Figure S2c).

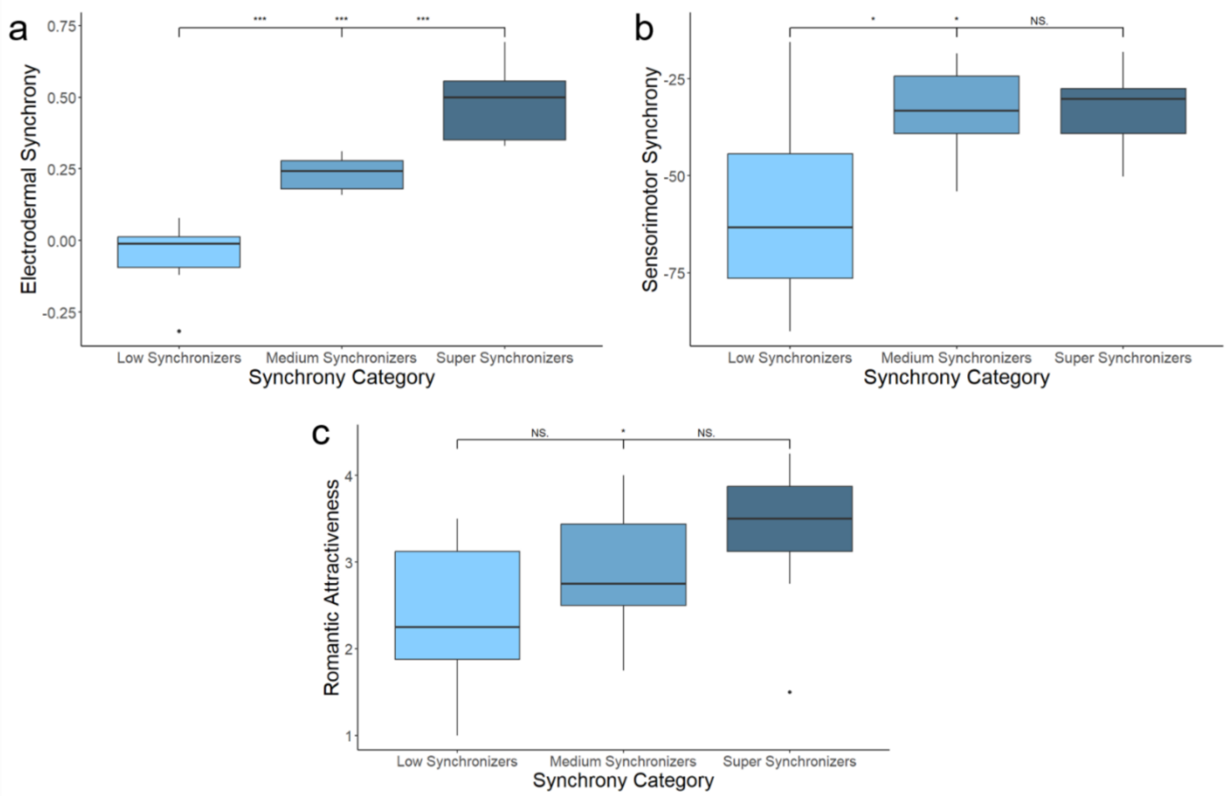

Figure S2. Synchrony and attraction in the different synchrony categories. (a) Super Synchronizers, Medium Synchronizers, and Low Synchronizers differ in their electrodermal synchrony scores. (b) Low Synchronizers differ from Medium Synchronizers and Super Synchronizers in sensorimotor synchrony scores. (c) Low Synchronizers differ from Super Synchronizers in romantic attractiveness scores. Synchrony groups are based on the participant's electrodermal synchrony scores. Super Synchronizers (Dark Blue, N=11), Medium Synchronizers (Medium Blue, N=10), and Low Synchronizers (Bright Blue, N=11). The lower and upper hinges correspond to the 25th and 75th percentiles, while the lower and upper whiskers extend to the smallest and largest values (data points beyond the end of the whiskers are outliers). * represents p-values between 0.01 and 0.05; *** represents p-values smaller than 0.001.

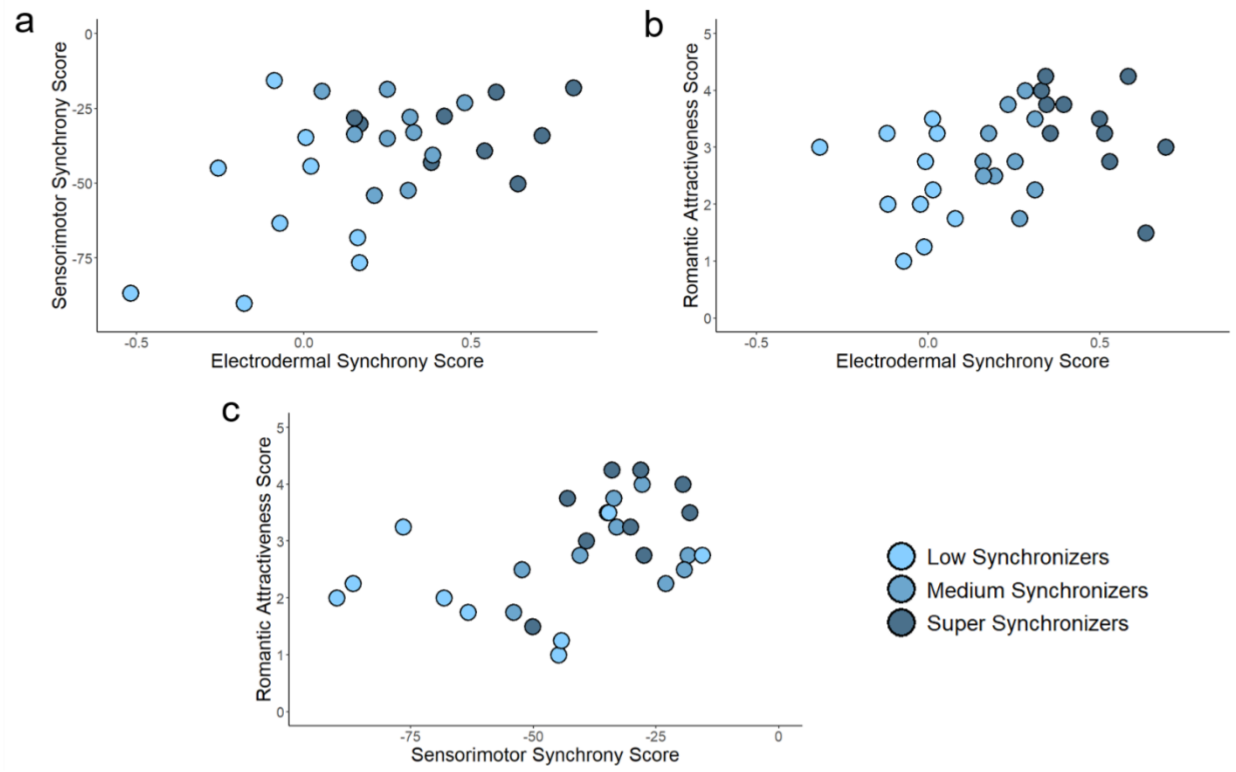

Figure S3. The relationships between electrodermal synchrony, sensorimotor synchrony, and romantic attractiveness in the different synchrony groups. These scatterplots show the associations between electrodermal synchrony, sensorimotor synchrony, and romantic attractiveness in the different synchrony categories. Super Synchronizers (Dark Blue, N=11), Medium Synchronizers (Medium Blue, N=10), and Low Synchronizers (Light Blue, N=11). Participants are divided into three synchrony groups based on their electrodermal synchrony scores, and darker blue represents a higher synchrony level.

Supplementary Note S4: Electrodermal synchrony and mutual attraction

To assess the association between synchrony and attraction at the level of the date, we conducted a multilevel model analysis on 64 dates, testing the extent to which electrodermal synchrony during the date is associated with increased mutual attraction at

the end of the date. While significant in previous work from our lab[8], this analysis did not converge in a multilevel model. The Spearman correlation values are: Synchrony during the first 2 minutes and mutual attraction: *Spearman r* = 0.081; *p* = 0.527; *95% confidence interval* = [-0.163, 0.324]; N = 64 dates. Synchrony during the entire date and mutual attraction: *Spearman r* = 0.231; *p* = 0.066; *95% confidence interval* = [-0.003, 0.464]; N = 64 dates.

Supplementary References

1. Abu Salih, M. *et al.* Evidence for cultural differences in affect during mother–infant interactions. *Sci Rep* **13**, 4831 (2023).
2. Miles, L. K., Nind, L. K. & Macrae, C. N. The rhythm of rapport: Interpersonal synchrony and social perception. *Journal of Experimental Social Psychology* **45**, 585–589 (2009).
3. Hirsch, J., Zhang, X., Noah, J. A. & Ono, Y. Frontal temporal and parietal systems synchronize within and across brains during live eye-to-eye contact. *NeuroImage* **157**, 314–330 (2017).
4. Gumilar, I. *et al.* Inter-brain Synchrony and Eye Gaze Direction During Collaboration in VR. in *CHI Conference on Human Factors in Computing Systems Extended Abstracts* 1–7 (ACM, New Orleans LA USA, 2022). doi:10.1145/3491101.3519746.
5. Kinreich, S., Djalovski, A., Kraus, L., Louzoun, Y. & Feldman, R. Brain-to-Brain Synchrony during Naturalistic Social Interactions. *Sci Rep* **7**, 17060 (2017).
6. Cappella, J. N. Behavioral and judged coordination in adult informal social interactions: Vocal and kinesic indicators. *Journal of Personality and Social Psychology* **72**, 119–131 (1997).

7. Grammer, K., Honda, M., Juette, A. & Schmitt, A. Fuzziness of nonverbal courtship communication unblurred by motion energy detection. *Journal of Personality and Social Psychology* **77**, 487–508 (1999).
8. Zeevi, L. *et al.* Bio-behavioral synchrony is a potential mechanism for mate selection in humans. *Sci Rep* **12**, 4786 (2022).
