# Supplementary material for: Social and nonsocial synchrony are interrelated and romantically attractive
Source: Commun Psychol. 2024 Jun 10;2:57. doi: 10.1038/s44271-024-00109-1 (PMC11332061; doi:10.1038/s44271-024-00109-1)
Supplement: Supplementary file 3 — Reporting Summary [file 44271_2024_109_MOESM3_ESM.pdf]

nature portfolio

Corresponding author(s): Dr. Shir Atzil

Last updated by author(s): May 14, 2024



# Reporting Summary

Nature Portfolio wishes to improve the reproducibility of the work that we publish. This form provides structure for consistency and transparency in reporting. For further information on Nature Portfolio policies, see our Editorial Policies and the Editorial Policy Checklist.

## Statistics

For all statistical analyses, confirm that the following items are present in the figure legend, table legend, main text, or Methods section.

| n/a | Confirmed | |
|---|---|---|
| ☐ | ☒ | The exact sample size (*n*) for each experimental group/condition, given as a discrete number and unit of measurement |
| ☐ | ☒ | A statement on whether measurements were taken from distinct samples or whether the same sample was measured repeatedly |
| ☐ | ☒ | The statistical test(s) used AND whether they are one- or two-sided<br>*Only common tests should be described solely by name; describe more complex techniques in the Methods section.* |
| ☒ | ☐ | A description of all covariates tested |
| ☐ | ☒ | A description of any assumptions or corrections, such as tests of normality and adjustment for multiple comparisons |
| ☐ | ☒ | A full description of the statistical parameters including central tendency (e.g. means) or other basic estimates (e.g. regression coefficient) AND variation (e.g. standard deviation) or associated estimates of uncertainty (e.g. confidence intervals) |
| ☐ | ☒ | For null hypothesis testing, the test statistic (e.g. *F*, *t*, *r*) with confidence intervals, effect sizes, degrees of freedom and *P* value noted<br>*Give P values as exact values whenever suitable.* |
| ☒ | ☐ | For Bayesian analysis, information on the choice of priors and Markov chain Monte Carlo settings |
| ☐ | ☒ | For hierarchical and complex designs, identification of the appropriate level for tests and full reporting of outcomes |
| ☐ | ☒ | Estimates of effect sizes (e.g. Cohen's *d*, Pearson's *r*), indicating how they were calculated |

*Our web collection on statistics for biologists contains articles on many of the points above.*

## Software and code

Policy information about availability of computer code

| | |
|---|---|
| Data collection | Qualtrics |
| Data analysis | R (2022.12.0+353) |

For manuscripts utilizing custom algorithms or software that are central to the research but not yet described in published literature, software must be made available to editors and reviewers. We strongly encourage code deposition in a community repository (e.g. GitHub). See the Nature Portfolio guidelines for submitting code & software for further information.

## Data

Policy information about availability of data

All manuscripts must include a data availability statement. This statement should provide the following information, where applicable:

- Accession codes, unique identifiers, or web links for publicly available datasets
- A description of any restrictions on data availability
- For clinical datasets or third party data, please ensure that the statement adheres to our policy

All data and code used in the analyses were uploaded to osf.org (at https://osf.io/nzhv6/) and can become available upon request.

## Human research participants

Policy information about studies involving human research participants and Sex and Gender in Research.

| | |
|---|---|
| Reporting on sex and gender | Gender has been determined based on self-report. In the speed-dating experiment, the participants' gender was crucial for the design, as each date included one man and one woman. In the online experiment gender was not relevant for the design. |
| Population characteristics | In the online experiment, one hundred sixty participants participated. Out of these, 16 participants were excluded for not meeting the inclusion criteria. The final dataset consists of 144 participants (76 women, 68 men), aged 18–30 (Mean = 24.89, SD = 3.61 years). All participants were Hebrew speakers.<br>In the speed-dating experiment, Forty-eight students (24 men, 24 women) aged 19–28 (Mean = 24.72, SD = 1.93 years) participated. All participants are native Hebrew speakers, not diagnosed with any psychiatric disorder, heterosexual, cis-gender, single, and interested in a romantic relationship. |
| Recruitment | In the online experiment, participants were recruited via iPanel. In the speed-dating experiment, participants were recruited via social media and the university's online experiment system. |
| Ethics oversight | The ethical committee of the Faculty of Social Sciences of the Hebrew University of Jerusalem approved the experiment per relevant guidelines and regulations. Each participant signed an informed consent form before participation. |

Note that full information on the approval of the study protocol must also be provided in the manuscript.

# Field-specific reporting

Please select the one below that is the best fit for your research. If you are not sure, read the appropriate sections before making your selection.

☐ Life sciences ☒ Behavioural & social sciences ☐ Ecological, evolutionary & environmental sciences

For a reference copy of the document with all sections, see nature.com/documents/nr-reporting-summary-flat.pdf

# Behavioural & social sciences study design

All studies must disclose on these points even when the disclosure is negative.

| | |
|---|---|
| Study description | Recent research shows that physiological synchrony is correlated with romantic attraction. Yet, it is still unknown whether synchrony can causally determine attraction. Here, we tested whether the ability to synchronize is an individual propensity that predicts attraction. In a preregistered online experiment, we discovered that manipulating the level of physiological synchrony between a man and a woman affects their attractiveness ratings by participants, suggesting a causal role for synchrony in determining attraction. In a naturalistic speed-dating lab experiment, we recorded the participants' social physiological synchrony while dating and nonsocial sensorimotor synchrony in a finger tapping task. We discover that the individual propensity to synchronize in social and nonsocial tasks is correlated. Some individuals synchronize better regardless of partners or tasks, and such Super Synchronizers are rated as more attractive. |
| Research sample | In the online experiment, 144 participants (76 women, 68 men) took part, and their ages were between 18–30 (Mean = 24.89, SD = 3.61 years). All participants were Hebrew speakers, and were representative of the Hebrew-speaking Israeli population in those ages.<br>In the speed-dating experiment, Forty-eight students (24 men, 24 women) aged 19–28 (Mean = 24.72, SD = 1.93 years) participated. All participants were native Hebrew speakers, not diagnosed with any psychiatric disorder, heterosexual, cis-gender, single, and interested in a romantic relationship. |
| Sampling strategy | In the online experiment, we estimated the target sample size, using power analysis (G*Power) for repeated measures MANOVA, between two different groups, with one independent variable (bio-behavioral synchrony), and two dependent variables (attractiveness of the actors, attraction between the actors). The parameters of the power analysis are a power of 0.95 to detect an effect size of Cohen's f=0.3 at a standard α=0.05 error probability rate, with an estimated correlation between repeated measures of r=0.8. The target sample size was 132 participants (66 participants per condition), and we recruited 160 participants.<br>In the speed-dating experiment, power was calculated according to the association between synchrony and romantic attractiveness. To estimate our target sample size, we used bootstrapped power calculation on a separate dataset of a previous speed-dating experiment from our lab with 30 participants. Using bootstrap sampling (n=10,000) to assess the power of different sample sizes, we found that to reach statistical power of 95%, a sample size of 48 participants is needed. Forty-eight participants were recruited, out of which physiological data were available for 32 participants (64 dates), with a statistical power of 81% in the analyses that include electrodermal synchrony. |
| Data collection | In the online experiment, participants observed a video with either a high or low synchrony interaction between a man and a woman, and then answered a set of questions regarding their attractiveness, using their computer or smart phone.<br>In the speed-dating experiment, participants met for five-minute speed-dates in a dedicated room with a homey arrangement, while their physiology was sampled at 4 Hz, using Empatica E4 wristbands. Before each date, participants rated their motivation to succeed in the date, and after the date, they rated their romantic attraction to the partner. The tapping task data was recorded via Audacity while participants tried to synchronize to the beat of an external metronome by tapping with their finger on a wooden box. |

| | |
|---|---|
| Timing | The online experiment's data collection started on September 8, 2022 and ended on September 12, 2022. The speed-dating experiment's data collection started on March 13, 2022 and ended on March 24, 2022. |
| Data exclusions | In the online experiment, out of 160 participants 15 reported a diagnosis of a psychiatric disorder, and one exceeded the predefined age range. In the speed-dating experiment, no data was excluded. |
| Non-participation | No participant dropped out or declined participation. |
| Randomization | In the online experiment, participants were randomly assigned to one of the two conditions: watch a high synchrony interaction (73 participants); or watch a low synchrony interaction (71 participants). In the speed-dating experiment, participants were not allocated into experimental groups. |

# Reporting for specific materials, systems and methods

We require information from authors about some types of materials, experimental systems and methods used in many studies. Here, indicate whether each material, system or method listed is relevant to your study. If you are not sure if a list item applies to your research, read the appropriate section before selecting a response.

## Materials & experimental systems

| n/a | Involved in the study | |
|---|---|---|
| ☒ | ☐ | Antibodies |
| ☒ | ☐ | Eukaryotic cell lines |
| ☒ | ☐ | Palaeontology and archaeology |
| ☒ | ☐ | Animals and other organisms |
| ☒ | ☐ | Clinical data |
| ☒ | ☐ | Dual use research of concern |

## Methods

| n/a | Involved in the study | |
|---|---|---|
| ☒ | ☐ | ChIP-seq |
| ☒ | ☐ | Flow cytometry |
| ☒ | ☐ | MRI-based neuroimaging |
